# Activation Mechanism of RhoA Caused by Constitutively Activating Mutations G14V and Q63L

**DOI:** 10.3390/ijms232415458

**Published:** 2022-12-07

**Authors:** Shiyao Chen, Zirui Zhang, Yijing Zhang, Taeyoung Choi, Yaxue Zhao

**Affiliations:** School of Pharmacy, Shanghai Jiao Tong University, 800 Dongchuan Road, Shanghai 200240, China

**Keywords:** RhoA, activating mutations, molecular dynamics, state transition, solvent exposure

## Abstract

RhoA, a member of Rho GTPases, regulates myriad cellular processes. Abnormal expression of RhoA has been implicated in various diseases, including cancers, developmental disorders and bacterial infections. RhoA mutations G14V and Q63L have been reported to constitutively activate RhoA. To figure out the mechanisms, in total, 1.8 μs molecular dynamics (MD) simulations were performed here on RhoA^WT^ and mutants G14V and Q63L in GTP-bound forms, followed by dynamic analysis. Both mutations were found to affect the conformational dynamics of RhoA switch regions, especially switch I, shifting the whole ensemble from the wild type’s open inactive state to different active-like states, where T37 and Mg^2+^ played important roles. In RhoA^G14V^, both switches underwent thorough state transition, whereas in RhoA^Q63L^, only switch I was sustained in a much more closed conformation with additional hydrophobic interactions introduced by L63. Moreover, significantly decreased solvent exposure of the GTP-binding site was observed in both mutants with the surrounding hydrophobic regions expanded, which furnished access to water molecules required for hydrolysis more difficult and thereby impaired GTP hydrolysis. These structural and dynamic differences first suggested the potential activation mechanism of RhoA^G14V^ and RhoA^Q63L^. Together, our findings complemented the understanding of RhoA activation at the atomic level and can be utilized in the development of novel therapies for RhoA-related diseases.

## 1. Introduction

The Rho GTPase family, comprised of Rho, Rac, and Cdc42, was discovered in the 1990s and is best known for its role in regulating cytoskeletal rearrangements [1], cell motility [2], cell polarity, axon guidance, vesicle trafficking and the cell cycle [3,4]. Most Rho GTPases cycle between inactive GDP-bound and active GTP-bound forms, therefore being considered molecular switches. Conformational changes between these two nucleotide-bound states are mainly localized to two regions known as a switch I and switch II, which are highly conserved among Rho family members [5,6,7,8]. The binary state cycle is regulated by three types of proteins [9]. Guanine nucleotide exchange factors (GEFs) catalyze the nucleotide exchange from GDP to GTP, thereby activating the GTPase [10,11], whereas GTPase-activating proteins (GAPs) inactivate it by increasing the intrinsic GTP hydrolysis rate of the GTPase [11]. Another mechanism of inactivating Rho GTPase is through guanine nucleotide dissociation inhibitors (GDIs). GDIs can sequester some of the Rho GTPases in their GDP-bound forms in the cytosol, thus preventing them from being activated by GEFs and blocking access to downstream targets [12,13].

**Figure 1:**
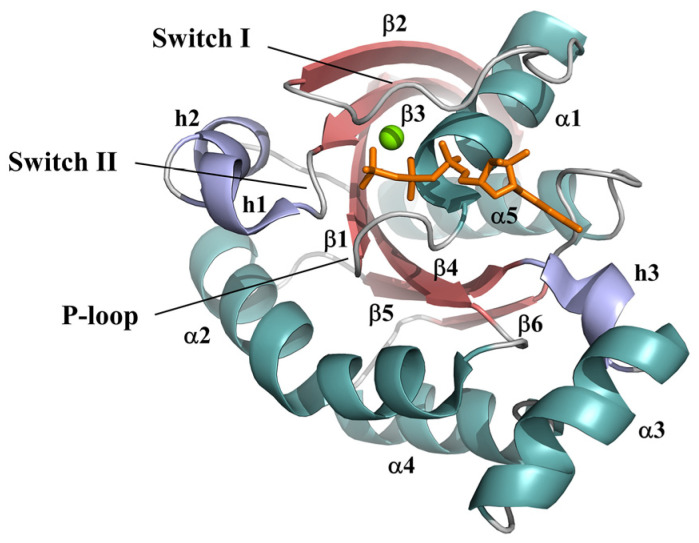
Crystal structure of GTPγS-RhoA^G14V^ (PDB ID: 1A2B) with β-strands (red), α-helices (teal), and 3_10_-helices (slate) differently colored and labeled. GTP-γS—orange stick; Mg^2+^ ion—green ball.

RhoA, one of the three most well-studied Rho GTPases (the other two are Rac1 and Cdc42) [14], belongs to the Rho branch of the Rho family. The overall structure of RhoA consists of a six-stranded β-sheet surrounded by five α-helices and three 3_10_-helices (Figure 1). As previously reported in other related small GTPases [5,15,16,17,18,19], it contains three functional regions, a phosphate-binding loop (P-loop; residues 12–19), switch I (residues 32–42), and switch II (residues 61–78), which are essential to the strong nucleotide binding. Moreover, the presence of the Mg^2+^ ion in the binding pocket has also been shown to be indispensable for both nucleotide binding and the GTPase activity of small GTPases [20,21]. In the RhoA-GTP complex, Mg^2+^ coordinates to oxygens from the triphosphate group of GTP together with adjacent RhoA residues, contributing to the binding stability of GTP and sustaining the whole structural conformation.

RhoA is activated in response to the binding of chemokines, cytokines, and growth factors and regulates actomyosin contractility and cell cycle progression [22,23]. Increasing evidence has associated elevated RhoA signaling with human cancer metastasis and progression [24,25], through increased RhoA expression [26,27,28], activating mutation or increased expression of GEFs [29,30,31], or from deletion or decreased expression of GAPs [32,33]. There are two common activating mutations of RhoA, G14V and Q63L, which are deficient in GTPase activity and thereby render the protein constitutively active. Notably, while the rate constant of in vitro GTP hydrolysis for these two mutants decreased similarly when compared with RhoA^WT^ (3.8 × 10^−6^ s^−1^, 5.1 × 10^−6^ s^−1^ and 100 × 10^−6^ s^−1^ for RhoA^G14V^, RhoA^Q63L^, and RhoA^WT^ respectively) [8], differences were found in their other functional properties. For example, an altered morphology was observed in transformed rat fibroblasts transfected with RhoA^Q63L^ but not in the G14V mutant [34]. Another study of mammalian cells addressed differential subcellular localization of the two mutants, with remarkable differences noted in their ability to bind to GDIs [35]. Over the past decades, these two mutants have often been utilized to study the signaling and cellular activities of RhoA, providing important findings, such as the ability of RhoA to regulate the formation of actin stress fibers and coordinate the actin cytoskeleton during cell adhesion and migration [36,37], which have an important role in guiding follow-up studies.

The molecular activation mechanism of RhoA and its constitutively activated mutants have attracted significant research from researchers. In 2017, Kumawat, A. et al. revealed the existence of multiple conformational intermediate states of switch I region in RhoA’s nucleotide-dependent switching based on molecular dynamics (MD) simulations, nd proposed that the favorable hydrogen bond interactions contributed by the γ-phosphate of GTP can counterbalance the energetic penalty of the highly exposed hydrophobic groups in their GTP-bound form [38]. Similar works on other Rho family GTPases, such as Rac1 and Cdc42, have also been reported more recently. In Rac1, the constitutively activating mutation Q61L (equivalent to Q63L in RhoA) and oncogenic mutation Y72C were found to affect the intrinsic dynamics underlying the binding of Rac1 to GTP and its downstream effector PAK1, leading to more flexible GTP and PAK1-binding residues on switch I and II [39]. As for Cdc42, its insert region (Cdc42 residues 122–135) was observed to exhibit much higher conformational fluctuations when binding with GDP than GTP in both of the wild-type Cdc42 and its G12V and Q61L (equivalent to G14V and Q63L in RhoA) mutants, which may account for the decrease in the ability of the GDP form to bind to effectors [40]. However, despite the efforts to date, the detailed activation mechanism of RhoA^G14V^ and RhoA^Q63L^ still remains unclear.

Here, we aim to investigate the molecular basis of the constitutive activation of RhoA caused by G14V and the Q63L mutation. Both sites are located in the nucleotide-binding pocket and interfere with the hydrolysis of the γ-phosphate of GTP. A total of 1.8 μs explicit MD simulations were performed on the wild-type RhoA and the two mutants G14V and Q63L in their GTP-bound forms, followed by a dynamic analysis to investigate the structural and dynamical properties of each model. We found that both mutations dramatically affected the conformational dynamics of the switch regions, especially switch I, resulting in varying degrees of state transition from inactive (the dominant form observed in RhoA^WT^-GTP) to active. These changes tightened the binding between GTP and the mutants, as reflected in the increased values of the binding free energy. We also showed that the mutations decreased the exposure of the nucleotide-binding site to the solvent, which made it much more difficult to access the essential water molecules required for GTP hydrolysis. Collectively, our findings increased the comprehension of the G14V- and Q63L-caused RhoA activation and may provide a valuable foundation for future drug development targeting RhoA and other RhoA-related small GTPases.

## 2. Results

### 2.1. Different Conformational Dynamics of the Switch Regions between the Wild-Type GTP-Bound RhoA and the Mutants

We performed explicit MD simulations on the wild-type RhoA and its G14V and Q63L mutants in their GTP-bound forms. Each system was submitted to triple-parallel 200 ns simulations, with a total timescale of 1.8 μs. For each trajectory, the root mean square deviations (RMSDs) of the backbone atoms of RhoA along the simulation time were calculated to monitor the convergence and stability (Figure S1). During the simulations, significant differences were observed among the three models in the conformational and dynamical behaviors of RhoA, especially the two switch regions.

**Figure 2:**
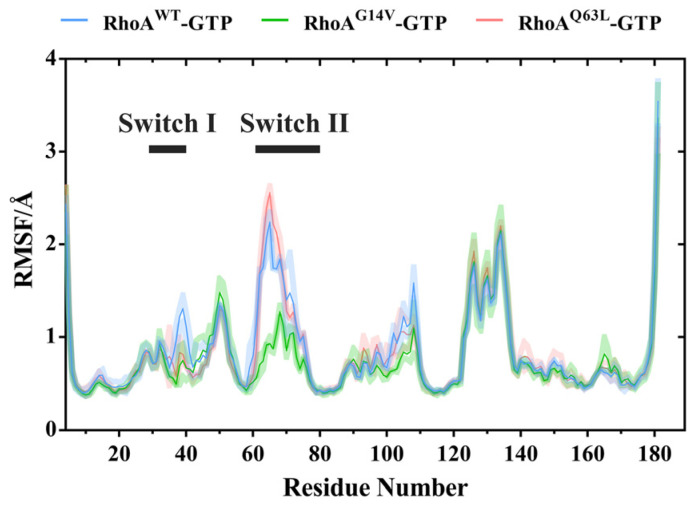
The RMSFs for each residue of RhoA in the complexes of RhoA^WT^-GTP (blue), RhoA^G14V^-GTP (green), and RhoA^Q63L^-GTP (red). All the frames were first aligned to the initial frame before calculating. Averages were obtained from the triple-independent simulation runs, and error bars correspond to the standard deviation.

To monitor the fluctuations of the individual residues during simulation, we first measured the root mean square fluctuations (RMSFs) for each amino acid of RhoA throughout the simulations (Figure 2). In general, both mutants showed fewer fluctuations than the wild type, whereas the two regions with the most distinct changes corresponded roughly to switch I and II. For the G14V mutant, the RMSFs for both of its switch regions were significantly lower than that found in the wild-type system, indicating an obvious decrease in conformational flexibility. For the Q63L mutant, its switch I region also exhibited much lower RMSFs, while some of its switch II region residues, residues 62–67, exhibited slightly higher RMSFs when compared with other systems, which possibly suggested distinct conformational behaviors caused by the two mutations. Besides, the C-terminal region of α2 helix (residues 95–110) showed varying degrees of lowered RMSFs in the two mutated systems. Considering that in the tertiary structure, this region is located next to switch II (Figure 1), the decrease in its fluctuations may also reflect, to some extent, the different conformational dynamics of switch II in the mutants.

**Figure 3:**
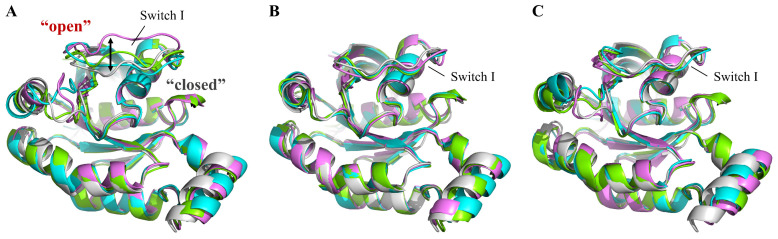
Superimposition of the top-three cluster conformations (cluster1—green; cluster2—cyan; cluster3—violet) of GTP-bound wild-type RhoA (**A**) and the two mutants G14V (**B**) and Q63L (**C**). The initial conformations for each system are also shown here as a gray cartoon.

Cluster analysis mainly showed three conformations of the switch I region in the simulations among all three systems: open, semi-open, and closed (Figure 3 and Figure S2). As shown in Figure 3A, the switch I region of RhoA^WT^-GTP preferred relatively open conformations. Two of the top three clusters, cluster 1 (green, semi-open) and 3 (violet, open), appeared in 46% and 18% of the conformations, respectively, making up 64% in total (Figure S2). However, in both RhoA^G14V^-GTP and RhoA^Q63L^-GTP (Figure 3B,C), the conformation of switch I was entirely restricted to be closed in all the centroid structures obtained from clustering. Moreover, compared with the wild type, the C-terminal region of switch I (residues 40–42) formed additional β-sheet structures in the two mutant systems (Figure 3 and Figure S3), which was consistent with the observed decrease in conformational flexibility. In the case of the switch II region, the G14V mutant exhibited the most stable switch II conformation, with the N-terminal of switch II much closer to the GTP-binding site (Figure 3).

### 2.2. State Transition of the GTP-Bound RhoA Induced by Mutation

**Figure 4:**
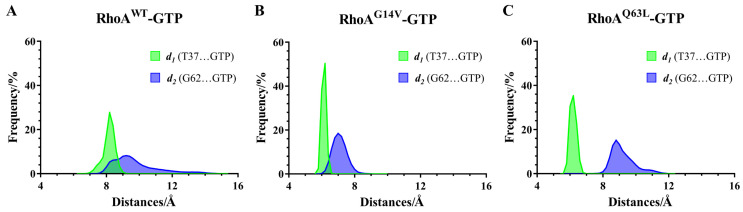
The probability distributions of two atom-pair distances, *d*_1_ and *d*_2_, were calculated on the snapshots obtained from the triple independent runs of the complexes of RhoA^WT^-GTP (**A**), RhoA^G14V^-GTP (**B**), and RhoA^Q63L^-GTP (**C**).

Previous studies of other GTPase family members like Ras [41,42,43,44] and Cdc42 [45] proposed and validated the existence of two interconverting conformations in their GTP-bound forms, namely inactive and active states. The active state exhibited a tightly closed conformation of switch I and II, characterized by two highly conserved hydrogen bonds formed by GTP’s γ-phosphate to T35 (T37 in RhoA) and G60 (G62 in RhoA), whereas the inactive state contained three substates 1, 2, and 3, described by the loss of both γ-phosphate-mediated interactions with T35 and G60, the loss of γ-phosphate-T35 interaction only, and the loss of γ-phosphate-G60 interaction only, respectively [43]. The populations of the states varied from the GTPases. Higher populations of the inactive state were discovered to show higher dissociation and association rate constants for GTP, which suggested a possibility that nucleotide-free small GTPases may preferentially yield the inactive state after association with GTP and then convert to the active state for effector binding [46]. Here, to further explore and quantify the conformational changes of RhoA-GTP caused by the two dominantly active mutations, we tried to delineate RhoA conformations by the inactive and active states. Two atom-pairs distances were defined, one from the C_α_ atom of switch I residue T37 to the GTP P_β_ atom (*d*_1_) and the other from the C_α_ atom of switch II residue G62 to the GTP P_β_ atom (*d*_2_), and their individual probability distributions in the last 120 ns of the simulations for each system were calculated and plotted (Figure 4).

As shown in Figure 4A, in the RhoA^WT^-GTP system, the probability distribution of *d*_1_ can be roughly divided into two intervals, one with values from 6.8 to 7.8 Å, and the other from 7.8 to 9.2 Å. Representative structure analysis corresponding to the two *d*_1_ intervals indicated that in the former structures, the characteristic T35-γ-phosphate hydrogen bond interaction could still be observed in a few snapshots, while it disappeared in the latter interval. Meanwhile, the distribution of *d*_2_ was more scattered between 7.8 to 15.0 Å, with the highest statistical frequency even lower than 10%, suggesting the loss of the G60-mediated interactions with GTP over the statistical periods. Collectively, our data suggested that the wild-type GTP-bound RhoA mainly existed in the inactive substate 1 and 3 in solution, where the inactive substate 1 conformation made up the vast majority.

We next investigated the impacts of the mutations on the conformational ensemble of RhoA-GTP. Compared to the plot of RhoA^WT^-GTP, the probability distribution of *d*_1_ in both mutants (Figure 4B,C) wase confined to a new interval with the values from approximately 5.6 to 7.0 Å, suggesting the formation of additional hydrogen bond between GTP and T37 caused by the mutations. Differences occurred in the probability distribution of *d*_2_. In RhoA^G14V^-GTP, most *d*_2_ values concentrated within the emergence of a new interval between 6.0 to 8.0 Å, indicating the potential characteristic G62-γ-phosphate interaction, while in RhoA^Q63L^-GTP, the values were in the interval greater than 8.0 Å but showed a significantly decreased mean and standard deviation in comparison with the wild type. Accordingly, though both mutants exhibited significant changes in the conformational preference of RhoA-GTP, G14V induced a completely inactive-to-active state transition, whereas Q63L merely shifted the majority of the ensemble from inactive substate 1 to inactive substate 3.

### 2.3. Significantly Increased Binding Affinity of GTP to Mutants G14V and Q63L

The binding free energies (BFEs) of GTP complexed with RhoA^WT^, RhoA^G14V^, and RhoA^Q63L^, including the total energies and the decomposed contributions per residue, were calculated using the MM/GBSA method. As listed in Table 1, the total BFEs of the two mutant systems were much lower than that of the wild type with differences more than 30 kcal mol^−1^, indicating significantly increased binding affinity between GTP and RhoA^Mut^, while mutant G14V exhibited the highest GTP-binding affinity with a total BFE of −290.72 ± 0.55 kcal mol^−1^.

Decomposed BFEs on each residue and the Mg^2+^ ion revealed more details (Table 1). A total of seven residues, as listed in Table 1, contributed more than 5 kcal mol^−1^ energy to the binding with GTP, where six were located on the P-loop, and the other was (Y34) located on switch I region, demonstrating the prominent role of P-loop in GTP binding. Among the six P-loop residues, the energy contribution of K18 was the most, which was more than 30 kcal mol^−1^ in all three systems and further increased in value after mutation. Besides, a mutation from glycine to valine nearly tripled the BFE of residue 14, from −5.43 ± 0.20 kcal mol^−1^ in RhoA^WT^ to −13.46 ± 0.04 kcal mol^−1^ in RhoA^G14V^. By contrast, changes in the energy contribution of the switch I residue Y34 caused by mutation were relatively small, with the largest disparity less than 3 kcal mol^−1^. The most striking changes happened on the Mg^2+^ ion. In RhoA^WT^-GTP, the decomposed BFE of Mg^2+^ was −30.59 ± 1.75 kcal mol^−1^, whereas in RhoA^G14V^-GTP and RhoA^Q63L^-GTP, it sharply increased to −77.00 ± 0.88 and −67.04 ± 1.92 kcal mol^−1^, respectively, serving as the main cause of the increase in total BFE value in both of the two mutants. Accordingly, Mg^2+^ was inferred to mediate essential interactions in the binding of RhoA with GTP, which would undergo critical changes after mutation.

### 2.4. Interactions between RhoA and GTP around the Nucleotide-Binding Site

**Figure 5:**
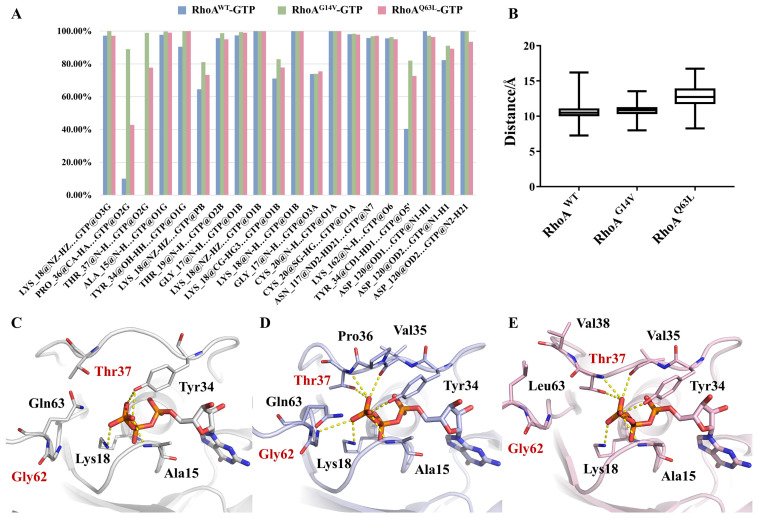
Interactions between GTP and RhoA^WT^, RhoA^G14V^, and RhoA^Q63L^. (**A**) Fractions of hydrogen bonds formed between GTP and RhoA. The fractions shown here are averaged over each system’s last 120 ns of the triple runs. (**B**) Distances from the C_δ_ atom of Q63 (or L63) to the P_β_ atom of GTP in different systems during the simulations. (**C**–**E**) Close-up on the nucleotide-binding site of complexes RhoA^WT^-GTP (**C**, gray), RhoA^G14V^-GTP (**D**, light blue), and RhoA^Q63L^-GTP (**E**, light pink). All the structures used here are the middle structures of the most populated clusters of each system obtained by clustering analysis. Crucial residues are labeled and shown as sticks (colored by element), with the γ-phosphate-mediated hydrogen bonds indicated as yellow dotted lines.

The molecular basis of the interactions between RhoA and GTP was focused on here to help explain the conformational changes of RhoA and the increase in binding stability with GTP occurring in the mutations. The fractions of hydrogen bonds between RhoA (wild type and the two mutants) and GTP were calculated over the last 120 ns of the corresponding triple runs, and the hydrogen bonds with an averaged fraction of more than 75% in any of the three systems were summarized and compared (Figure 5A; Table S1). In all systems, the α- and β-phosphate oxygens, as well as the guanine skeleton of GTP, formed highly stable hydrogen bond interactions with RhoA, in particular with P-loop residues G17, K18, T19, and adjacent C20. A moderate increase was observed in the fraction of the hydrogen bond formed between the O5′ atom and the switch I residue Y34, which was lower than 50% in the wild-type system but increased to 82% and 73% in the two mutated systems, partly contributing to the sustain of a more closed switch I conformation.

The main differences were in the γ-phosphate-mediated hydrogen bonds. In the RhoA^WT^-GTP system, as we stated before, the characteristic γ-phosphate-T37 hydrogen bond was barely observable in the simulations (Figure 5A,C; Table S1). However, this interaction kept highly stable in RhoA^G14V^-GTP and RhoA^Q63L^-GTP (Figure 5D,E; Table S1), with an average fraction of 99% and 78%, respectively. In addition, compared with Q63L, the G14V mutant exhibited two more unique hydrogen bonds. One of the bonds was formed by a γ-phosphate oxygen to the backbone of the switch I residue P36, of which the fraction was up to 89% for RhoA^G14V^-GTP but sharply decreased in the other two systems. The other was the characteristic γ-phosphate-G62 hydrogen bond. As shown in Figure 5D, the formation of this additional interaction significantly shortened the distance between the N-terminal of switch II and the nucleotide, confining the switch II region to the closed state. There were also some changes observed in the interactions between γ-phosphate and Y34. In RhoA^WT^-GTP, the side-chain phenolic hydroxyl hydrogen of Y34 formed two hydrogen bonds with two of the three γ-phosphate oxygens (Figure 5C), whereas in the two mutants, the strength of the Y34-O5′ interaction as mentioned above pulled Y34 to a conformation that covered GTP, leaving only one Y34-γ-phosphate hydrogen bond (Figure 5D,E).

We also measured the distance from the C_δ_ atom of Q63 (or L63 in the Q63L mutant) to the GTP P_β_ atom to monitor the side-chain orientation. As shown in Figure 5B, in both GTP-bound RhoA^WT^ and RhoA^G14V^, Q63 maintained a rather stable distance of about 10.0 to 11.0 Å from the nucleotide group. In the Q63L mutant, the distance of the CD1 atom of L63 wildly fluctuated between greater extremes with an average of up to 12.8 Å, indicating the loss of a crucial interaction. Together with the position and orientation of the side-chain amino group of Q63 observed in the representative structures (Figure 5C,D), we suggested that Q63 formed constant electrostatic interaction with the negatively charged γ-phosphate in both wild-type and G14V-mutated systems, which disappeared after the Q63L mutation. However, the mutation of Q63 to L63 at the same time introduced additional hydrophobic interactions with the N-terminal region of switch I (Figure 5E and Figure S4). As shown in Figure S4A, the distances from the mass centroid of the hydrophobic side chain of L63 to that of V38 in the Q63L mutant were stable at around 5.0 to 6.0 Å during the simulations, indicating potential hydrophobic interactions formed directly by L63 with V38. Moreover, compared to the wild type, the identically defined distances between the adjacent Y66 and V38 significantly decreased and stabilized after mutation (Figure S4B), which suggested newly formed interactions between the original hydrophobic switch residues induced by mutation. These hydrophobic interactions together helped to sustain the closed switch I conformation in the Q63L mutant, thereby strengthening the binding of GTP indirectly and offsetting the electrostatic-interaction loss to some extent.

**Figure 6:**
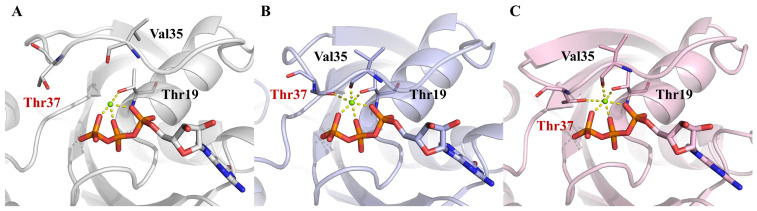
The coordination of Mg^2+^ ion (green ball) in RhoA^WT^-GTP (**A**, gray), RhoA^G14V^-GTP (**B**, light blue), and RhoA^Q63L^-GTP (**C**, light pink). The coordination interactions are indicated by dotted lines, and the residues involved are labeled and shown as sticks (colored by element).

Additionally, the coordination of the Mg^2+^ ion was crucial in both GTP binding and the conformational transition of mutated RhoA, which was in agreement with the remarkable changes observed in the decomposed binding free energy of Mg^2+^ before and after mutation. The position of the Mg^2+^ ion was virtually identical in the structures among all three systems, mainly stabilized by four highly conserved coordination bonds with the three GTP phosphate groups as well as the P-loop residue T19 (Figure 6). Interestingly, in RhoA^G14V^-GTP and RhoA^Q63L^-GTP, two more residues, V35 and T37, from the switch I region participated in Mg^2+^ coordination, constituting a typical hexahedral coordination sphere (Figure 6B,C), while in RhoA^WT^-GTP they were not in a position to coordinate Mg^2+^ (Figure 6A). These additional coordination interactions mediated by Mg^2+^ further brought together switch I and the nucleotide-binding site, in addition to accounting for the observed changes in switch I’s orientation in the two mutated systems.

### 2.5. Changes in the Exposure of the GTP-Binding Site

**Figure 7:**
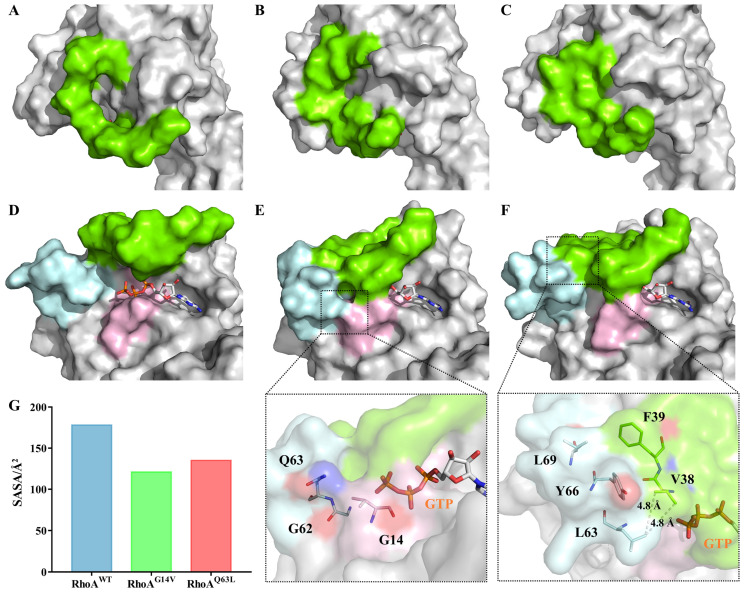
Differences in the protein surface properties between the wild-type RhoA and the mutants. (**A**–**F**) Surface of the three functional regions switch I (green), switch II (cyan), and P-loop (pink) in RhoA^WT^-GTP (**A**,**D**), RhoA^G14V^-GTP (**B**,**E**), and RhoA^Q63L^-GTP (**C**,**F**). In (**E**,**F**), the regions where the mutants cause critical changes are focused and magnified, with the key residues labeled and shown as sticks. All structures used here are extracted from the final snapshots (200 ns) of the MD trajectories for each system. (**G**) The SASA (Å^2^) of GTP in RhoA^WT^, RhoA^G14V^, and RhoA^Q63L^ averaged over the last 120 ns of corresponding triple runs.

Because any enzymatic mechanism of GTP hydrolysis [47,48,49], such as that proposed by Cavalli, A. et al. for the Cdc42/Cdc42GAP complex [47], primarily relies on the interactions and stabilization of essential water molecules in the active site, we compared the solvent accessibility of the GTP-binding site in RhoA^WT^ and its mutants. Significant changes were observed in both the protein surface shape and hydrophobicity. As shown in Figure 7A–F, the nucleotide-binding site was in much more open conformation in the RhoA^WT^-GTP than in the G14V and Q63L mutants, with the triphosphate groups of GTP almost completely exposed. We can also observe a hole constituted by the surface of switch I in the wild type (Figure 7A), which turned out to be blocked after mutation (Figure 7B,C). Notably, though the triphosphate groups of GTP in both mutants were buried inside the pocket formed by switch I and II, and the P-loop region, more changes occurred around the pocket surface that differed between the two systems.

In the G14V mutant, the substitution of valine for glycine introduced significantly increased steric hindrance. The bulky side chain of V14 made contact with G62 and Q63, joining the surface of the P-loop with switch II (Figure 7E). The far end of the tunnel for phosphate binding was thereby blocked, making it hard for the nucleophilic water molecule to attack from this direction. As for the Q63L mutant, the hydrophobic side chain of L63 formed additional interactions with the switch I residues V38 and F39, constituting a wide hydrophobic area with switch II residues Y66 and L69 (Figure 7F). This change in surface hydrophobicity would most likely disturb the original interaction pattern of the nucleophilic water molecule and prevent it from stably existing in the active site, thus impairing the GTP hydrolysis. This was further substantiated with the analysis of the solvent-accessible surface area (SASA, Å^2^) of GTP and the average number of water molecules around the GTP-hydrolyzing catalytic site in each system (Figure 7G and Figure S5). As shown in Figure 7G, the average SASA of the GTP in the wild-type system was 178.17 Å^2^, which decreased by approximately 32% and 24%, respectively, to 121.17 Å^2^ in RhoA^G14V^ and 135.12 Å^2^ in RhoA^Q63L^. Consistently, in Figure S5, mutant G14V exhibited the lowest average number of the catalytic site water molecules, approximately two, while such water molecule numbers in the mutant Q63L fluctuated to approximately three—both were lower than that in the wild type (five). G14V and Q63L caused decreases in the solvent exposure of the nucleotide-binding site and expanded hydrophobic regions, which hindered the access to water molecules required for hydrolysis and suggested a possible mechanism for the decrease in GTP hydrolysis activity in the mutants.

## 3. Discussion

**Figure 8:**
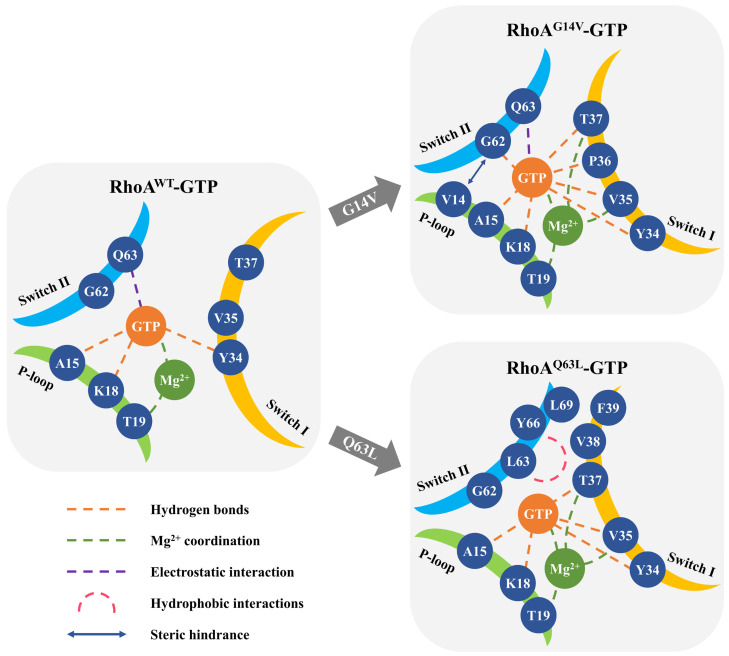
Schematic of the proposed activation mechanism of RhoA mutations G14V and Q63L.

In this work, MD simulations were combined with dynamic analysis to investigate the potential mechanism through which mutations G14V and Q63L affect the structural and dynamic behaviors of RhoA and ultimately result in constitutive activation. Unlike H-Ras [42] and K-Ras [43], as reported previously, the wild-type RhoA was observed to predominantly exist in an inactive state in its GTP-bound form, according to our study, despite sharing significant structure and sequence homology. Both characteristic hydrogen bonds formed by the γ-phosphate oxygens with T37 and G62 were lost in the corresponding structures, resulting in a relatively open site for GTP binding with large conformational flexibility. The two mutant systems, however, differed significantly from the wild type in the dynamics of the structures, especially in the switch regions, which confined the ensemble to much more closed conformation with increased binding affinity to GTP and concurrently decreased solvent exposure of the nucleotide-binding site, suggesting a possible activation mechanism as shown in Figure 8.

Generally, G14V and Q63L caused a similar inactive-to-active state transition in the switch I region, where the highly conserved T37 residue and the Mg^2+^ ion played an important part (Figure 8). Among Rho and Ras GTPases, T37 (T35 in some other GTPases like Cdc42 and Ras) has been widely shown to mediate a critical hydrogen bond from its main chain NH group to γ-phosphate, which serves as the trigger for the switch I conformational change [20]. In addition, T37 is required to bind RhoGAP, as an essential element in the catalysis of GTP hydrolysis by RhoA [50]. Here, we found that while the T37 and γ-phosphate mediated interaction was surprisingly absent in RhoA^WT^-GTP, it was highly stable throughout the simulations of both RhoA^G14V^-GTP and RhoA^Q63L^-GTP, thus triggering the switch I region of the two mutants changing from the inactive to the active states and largely contributing to the conformational stabilization. Moreover, as it flipped inside to form a hydrogen bond with GTP, T37 was also involved in Mg^2+^ coordination in RhoA^Mut^ via its side chain together with another switch I residue V35, by which it could further correlate with residues on the P-loop region. These additional interactions introduced by G14V and Q63L through allosteric pathways together shifted switch I to the closed active conformation, thereby more favorable for downstream effector binding and partly accounting for the mutation-dependent activation of RhoA protein.

The major differences between the activation of the G14V and Q63L mutants were concentrated in the switch II region, including its conformational dynamics and the interactions mediated by it with the other two functional regions. In RhoA^G14V^-GTP, an additional hydrogen bond was formed from one of the γ-phosphate oxygen to the main chain NH group of G62, which was rarely observed in both of our simulated wild-type and Q63L mutated systems and directly resulted in the much more closed and stable switch II conformation in G14V. Based on this hydrogen bond and the presence of the γ-phosphate-T37 interaction, mutation G14V was inferred to thoroughly shift the RhoA ensemble from the inactive substate 1 to the active state, whereas the transition induced by mutation Q63L, by definition, was more often halted in the inactive substate 3. In accordance with the crystallographic data and the studies on RhoA’s GTP hydrolysis [7,8], we suggested that the energy for the different states of GTP-bound RhoA may follow the order as active state > inactive substate 3 > inactive substate 1, whereas the order of GTPase activity is reversed accordingly. The inactive substate 2 described in the conformation of Ras-GTP [43], however, has not been observed in RhoA yet. In addition, as for the changes taking place in the interactions between the functional regions, the bulky side chain of V14 largely increased the steric hindrance between the P-loop and the C-terminal of switch II, further burying the γ-phosphate of GTP away from the solvent and contributing to the restriction on switch II conformation. Mutation from Q63 to L63, though it disrupted the original electrostatic interactions with GTP, expanded the hydrophobic surface together with the adjacent residues Y66, L69, and the switch I residues V38 and F39, which acted synergistically on the orientation of switch I and blocked the access of the hydrolysis-required nucleophilic water molecule from another direction, and thus impaired GTP hydrolysis as well. Taken together, these unique dynamic properties of mutants G14V and Q63L may help explain their observed in vivo differences, providing new insight into the molecular basis for Rho GTPase activation.

Two factors have been associated with high gene mutation rates and cause potential diseases: (i) proximity to telomeres (<50 Mbp) and/or (ii) high adenine and thymine (A + T) content (>59%) [51,52,53,54]. For human *RhoA*, it shows proximity (49 Mbp) to its telomeres with an A + T content of 56%, which are both very close to the critical values. In addition, *RhoA* is potentially highly mutable as frequent *RhoA* mutations have been found in a wide variety of human cancers, especially in angioimmunoblastic T-cell lymphoma (53–71%) [55,56,57] and diffuse gastric cancer (14–25%) [58]. The most prevalent cancer-associated *RhoA* mutation G17V is also an activating mutation, which impairs RhoA’s GTP hydrolysis activity. However, the specific role of RhoA^G17V^ in cancer progression and maintenance are only beginning to be elucidated. 

Our work made a successful attempt to explore the constitutive mutation-caused RhoA activation at the atomic level, which can be utilized in future studies on RhoA for other mutations or physiological functions. Moreover, the amino acid sequences of RhoA encoded in different species share several similarities, with highly conserved P-loop and switch regions where the RhoA mutational hotspots are located (Figure S6A). Considering the sequence identity and similarity (Figure S6B), we believe chimpanzees, mice, and rats would be promising choices for novel animal models to reproduce the expected phenotypes as found in humans and to conduct further RhoA studies.

## 4. Materials and Methods

### 4.1. Preparation of Models

The initial structures of the three GTP-bound RhoA systems were modeled based on the crystal structure of the RhoA^G14V^-GTPγS complex (Protein Data Bank, PDB ID: 1A2B). The GTPγS molecule in 1A2B was replaced with GTP, from which we obtained the initial RhoA^G14V^-GTP model. After that, residue V14 mutated back to native G14 to build RhoA^WT^-GTP, and residue Q63 mutated to L63 to model RhoA^Q63L^-GTP. The three resulting structures were then entered into the Protein Preparation Wizard of Schrödinger Maestro (version 2020, LLC, New York, NY, USA) [59] to fill in the missing side chains or atoms, assign bond orders, create disulfide bonds, predict the protonation state of histidine residues using PROPKA at pH = 7.0, and release the potential steric clashes. To further prepare the structures for simulations in AMBER, the tLEaP module of the AMBER20 package [60] was called, and the ff14SB forcefield [61] and Generalized AMBER force field (GAFF) [62] were used for the protein and GTP, respectively. The parameters for GTP and Mg^2+^ ion were taken from the AMBER parameter database (http://amber.manchester.ac.uk/, accessed on 22 October 2021). Finally, the proteins were solvated in a cubic simulation box with TIP3P water molecules [63], the size of which was set to ensure a distance of at least 10 Å between the proteins and the box boundaries. Nine Na^+^ ions were added to each system to neutralize the charge and simulate physiological conditions.

### 4.2. Simulation Protocol

All simulations were performed using the AMBER20 package. First, each solvated system was subjected to a 5000-step energy minimization under a positional restraint of 200 kcal mol^−1^ Å^−2^, with the first 1000 steps using the steepest descent algorithm and the last 4000 steps using conjugate gradient algorithm, followed by another 5000-step minimization without any restraints. Next, the protein atoms in the minimized systems were again restrained under 200 kcal mol^−1^ Å^−2^ positional restraint, while the solvent was first heated from 0 K to 300 K (100 ps) by using the Langevin thermostat and then pressurized to 1 atm (100 ps) with the temperature keeping constant at 300 K. Finally, the restraint was removed, and the whole system was allowed to equilibrate at the production temperature of 300 K and pressure of 1 atm for another 100 ps. Throughout the above simulations, a numerical integration time step of 2 fs was used, and the nonbonded pair list was updated every five steps.

In the production runs, a total of 1.8 μs MD simulations were performed with NPT conditions (constant temperature of 300 K and pressure at 1 atm) using the PMEMD module. Each model was simulated for independent triple 200 ns, with an integration step of 2 fs set for the production simulations. The trajectories were recorded every 1000 steps (2 ps) for subsequent analysis. During the simulations, Particle Mesh Ewald (PME) method [64] was used to calculate the long-range electrostatic interactions, whereas an 8.0 Å cutoff was used for nonbonded interactions. Meanwhile, SHAKE [65] was employed to perform constraints on all covalent bonds involving hydrogen atoms to remove the bond stretching freedom.

### 4.3. MD Trajectory Analysis

The Cpptraj code of the AMBER20 package was used to analyze the MD trajectories.

#### 4.3.1. RMSD/RMSF Calculation

The RMSDs and RMSFs of the backbone atoms (CA, C, N) of RhoA were calculated by mass-weighted average to investigate the fluctuation of each system and individual residues during the entire triple 200 ns simulations. The first snapshot was set as the reference structure for each MD trajectory.

#### 4.3.2. SASA Calculation and Water Molecule Counting

The SASA of the GTP-binding site in each system was calculated using the LCPO algorithm [66] and averaged over trajectories. To further assess the presence of water molecules in the GTP-hydrolyzing catalytic site, water molecules within 3.0 Å around the γ-phosphate of GTP were counted by frame. For each system, the average number of water molecules was calculated over frames from the last 120 ns of corresponding trajectories, with its probability distribution calculated and plotted.

#### 4.3.3. Secondary Structure Assignment Analysis

Secondary structure propensities for residues in RhoA were calculated by the DSSP method [67] implemented in AMBER, which assigns secondary structure types for residues based on backbone amide (N-H) and carbonyl (C=O) atom positions. For each residue from a different system, the structural propensity was calculated and averaged over all of the frames from the entire triple 200 ns trajectories.

#### 4.3.4. Cluster Analysis

The cluster analysis was performed with the average-linkage hierarchical agglomerative (bottom-up) approach. Snapshots extracted from every 40 ps of the corresponding triple 200 ns trajectories were first superimposed with the reference structure using all CA atoms to remove overall rotation and transition. Then, the backbone CA atom RMSD of the snapshots were compared in pairs. The clustering would be stopped when either of the five clusters were reached.

#### 4.3.5. Interaction Analysis

A hydrogen bond was defined as being between an acceptor heavy-atom A, a donor hydrogen atom H, and a donor heavy-atom D, including both backbone and side-chain atoms. Hydrogen bonds with an A–D distance less than 3.5 Å and an A–HºD angle greater than 120° were considered to be formed. Fractions of hydrogen bonds (hydrogen bond occupancies during the simulation time) were calculated and averaged over the last 120 ns of the triple trajectories. For hydrophobic interactions, the distances between the centroid of the side-chain heavy atoms of the selected residues were calculated over the corresponding triple trajectories and plotted with simulation time as the x-axis. 

### 4.4. Binding Free Energy Calculation

The molecular mechanics of the generalized Born surface area (MM/GBSA), developed by Kollman, P.A. et al. [68,69], is one of the most well-known end-point free energy methods, which achieves a good balance between computational efficiency and accuracy. In this work, AMBER20 was used to perform the MM/GBSA calculation on the binding free energy between RhoA and GTP. For each trajectory, snapshots from every 40 ps of the last 120 ns simulations were extracted for calculation, with the counter ions and water molecules stripped artificially beforehand.

In the MM/GBSA approach, the free energy for binding of the ligand (*L*) to the protein receptor (*R*) to form the complex (*RL*),

(1) $$\Delta G_{binding}=G_{RL}-\left(G_{R}+G_{L}\right)$$

can be decomposed into contributions of the different interactions and expressed as [70]

(2) $$\Delta G_{binding}=\Delta H-T\Delta S=\Delta E_{MM}+\Delta G_{sol}-T\Delta S$$

in which

(3) $$\Delta E_{MM}=\Delta E_{int}+\Delta E_{ele}+\Delta E_{vdW}$$

(4) $$\Delta G_{sol}=\Delta G_{pol}+\Delta G_{nonpol}$$

(5) $$\Delta G_{nonpol}=\gamma \cdot SASA+b$$

where $\Delta E_{MM}$, $\Delta G_{sol}$, and −TΔS are the changes in the gas-phase molecular mechanics (*MM*) energy, solvation free energy, and conformational entropy upon ligand binding, respectively. $\Delta E_{MM}$ includes the changes in the internal energies $\Delta E_{int}$ (bond, angle and dihedral energies), electrostatic energies $\Delta E_{ele}$, and van der Waals energies $\Delta E_{vdW}$. $\Delta G_{sol}$ is the sum of polar contribution $\Delta G_{pol}$ and nonpolar contribution $\Delta G_{nonpol}$, where the former is calculated using the GB model and the latter is usually estimated by SASA. The change in conformational entropy, −TΔS, is usually calculated by normal-mode analysis [70] on a set of conformational snapshots taken from the MD trajectory. However, because of its high computational cost and the fact that we focused more on the relative changes rather than the exact binding free energy values among the different systems, the entropy contribution was neglected in this work.

## 5. Conclusions

Aberrations in the functional Rho GTPases, mainly in their switching action, have been implicated in a number of diseases, such as cancers, developmental disorders and bacterial infections, where they occur in the constitutively active state [71,72]. Here, using MD simulations and dynamic analyses, we investigated the structural and dynamic behaviors of RhoA and its constitutively activated mutants G14V and Q63L in their GTP-bound forms, with the aim to reveal the unique activation mechanism under these two mutations. We found that the wild-type RhoA predominantly existed in the inactive state conformation upon GTP binding, whereas G14V and Q63L shifted the ensemble towards different active-like states. With the formation of the two characteristic hydrogen bonds between the γ-phosphate and the highly conserved residues T37 and G62 located on switch I and II, respectively, mutant G14V achieved a completely inactive-to-active state transition and showed the highest affinity to GTP among the three simulated systems according to interaction and energy analysis. Mutant Q63L, however, showed significantly more notable conformational changes in the switch I region when compared with the wild type, as the side chain of the leucine introduced additional hydrophobic interactions with switch I and served as another trigger for the close of the switch I conformation, while the impacts on switch II conformation was relatively subtle. The structure adopted by the RhoA^Q63L^-GTP complex throughout the simulations, as well as in the final snapshots, corresponded to the inactive substate 3 by strict definition, exhibiting a highly stable γ-phosphate-T37 hydrogen bond but lacking strong interactions between GTP and switch II. Considering the sharply decreased GTP hydrolysis rates of both RhoA^G14V^ and RhoA^Q63L^, it is likely that the activation of RhoA, especially the changes in GTPase activity, relies more on the conformation of switch I rather than switch II. In addition, we observed a significant decrease in the solvent exposure of the GTP-binding site in both mutants, with expanded hydrophobic regions which were exposed on the surface of the protein. Similar changes were reported for GDP to GTP exchange in wild-type RhoA [38], suggesting that the increase in hydrophobicity around the nucleotide-binding site might be another important factor in RhoA activation and further facilitate effector recognition [73,74]. As the mutations G14V and Q63L further enlarged the changes in the solvent exposure of GTP and the hydrophobic residues, the approach of the required water molecules to the active site became much more difficult, thereby proving to be unfavorable for GTP hydrolysis and holding the mutants in a better conformation to interact with downstream effectors.

In summary, our study first elucidates the differential dynamics of GTP-bound RhoA^WT^, RhoA^G14V^, and RhoA^Q63L^, suggesting a possible activation mechanism for RhoA mutants G14V and Q63L at the atomic level. These findings provide valuable molecular insights into the structural and dynamical properties of RhoA and its constitutively active mutants and have potential implications for the future development of novel therapies for RhoA-related diseases.

## Figures and Tables

**Table 1:** Binding free energy (BFE) of GTP complexed with RhoA^WT^, RhoA^G14V^, and RhoA^Q63L †^.

Energy Term	RhoA^WT^-GTP	RhoA^G14V^-GTP	RhoA^Q63L^-GTP
BFE Total			
$\Delta G_{binding}$	−227.08 ± 0.25	−290.72 ± 0.55	−262.51 ± 1.83
BFE per Residue			
G14 (or V14)	−5.43 ± 0.20	−13.46 ± 0.04	−5.80 ± 0.18
A15	−7.19 ± 0.22	−7.77 ± 0.17	−7.66 ± 0.28
C16	−5.70 ± 0.07	−6.16 ± 0.11	−5.94 ± 0.31
G17	−7.82 ± 0.29	−8.27 ± 0.20	−8.14 ± 0.16
K18	−31.56 ± 0.54	−42.73 ± 0.08	−36.43 ± 0.65
C20	−7.95 ± 0.18	−7.94 ± 0.06	−7.95 ± 0.11
Y34	−11.14 ± 0.40	−13.14 ± 0.06	−12.06 ± 0.14
Mg^2+^	−30.59 ± 1.75	−77.00 ± 0.88	−67.04 ± 1.92

† All the data listed here are in kcal mol^−1^. The average and standard deviation are calculated over each system’s last 120 ns of the triple runs.

## Data Availability

The data presented in this study are available on request from the corresponding author.

## Supplementary Materials

The following supporting information can be downloaded: https://www.mdpi.com/article/10.3390/ijms232415458/s1.
