# Supplementary material for: Activation Mechanism of RhoA Caused by Constitutively Activating Mutations G14V and Q63L
Source: Int J Mol Sci. 2022 Dec 7;23(24):15458. doi: 10.3390/ijms232415458 (PMC9778661; doi:10.3390/ijms232415458)
Supplement: Supplementary file 1 [file ijms-23-15458-s001.zip › ijms-2027656-supplementary.pdf]

SUPPLEMENTARY MATERIAL

Activation Mechanism of RhoA Caused by Constitutively Activating Mutations G14V and Q63L

Shiyao Chen †, Zirui Zhang †, Yijing Zhang, Taeyoung Choi and Yaxue Zhao *

School of Pharmacy, Shanghai Jiao Tong University, 800 Dongchuan Road, Shanghai 200240, China

* Correspondence: yaxuezhao@sjtu.edu.cn

† These authors contributed equally to this work.

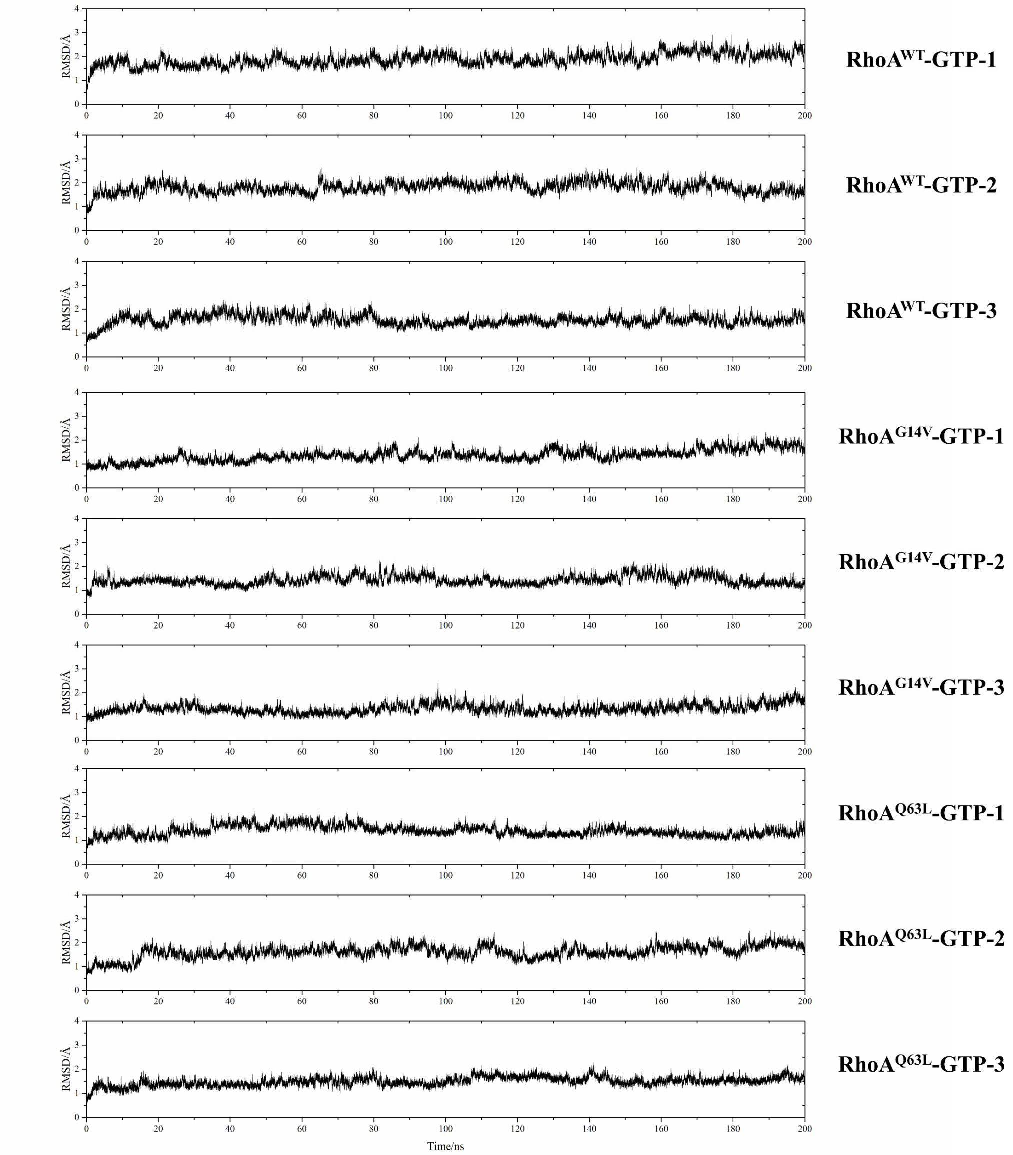

Figure S1. Time series of RMSD values of complexes $RhoA^{WT}$-GTP, $RhoA^{G14V}$-GTP, and $RhoA^{Q63L}$-GTP during the triple 200 ns MD simulations.

A **B** **C**

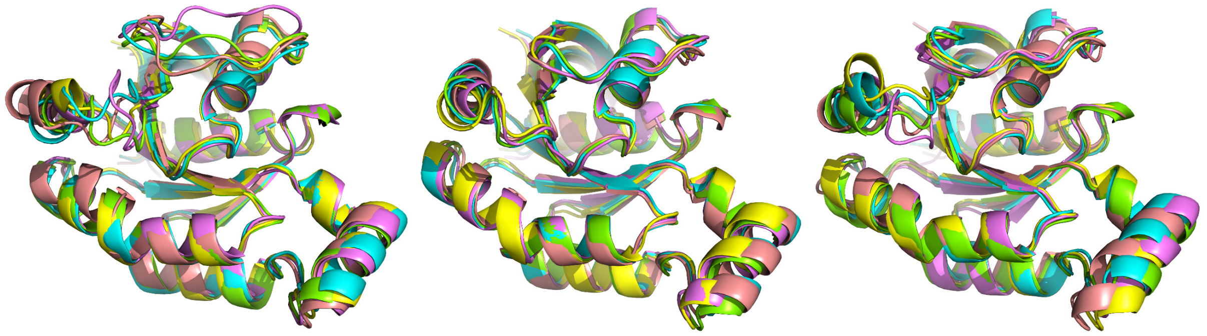

D

Cluster	Color	Fraction (%)		
		**RhoA^{WT}-GTP**	**RhoA^{G14V}-GTP**	**RhoA^{Q63L}-GTP**
1	Green	46.06	36.61	34.77
2	Cyan	24.06	34.94	28.92
3	Violet	17.75	19.35	24.97
4	Yellow	8.02	6.94	5.86
5	Salmon	4.10	2.16	5.47

Figure S2. Representative structures of the five clusters of RhoA in systems RhoA^{WT}-GTP (A), RhoA^{G14V}-GTP (B), and RhoA^{Q63L}-GTP (C) obtained from clustering. The colors and fractions of each cluster are listed in (D).

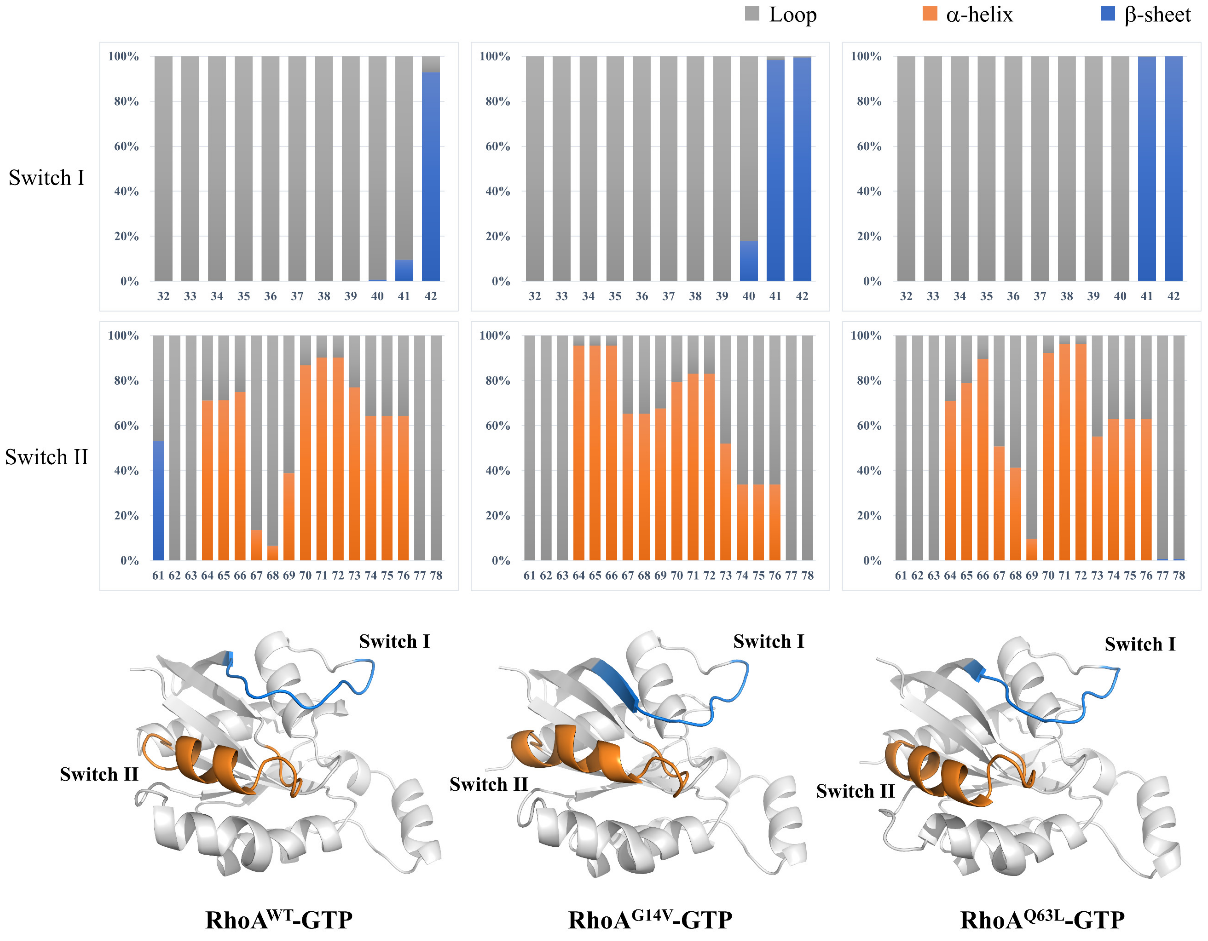

Figure S3. The overall secondary structure assignment for residues located on switch I and II regions in GTP-bound RhoA^{WT}, RhoA^{G14V}, and RhoA^{Q63L} throughout the MD simulations. The representative structure for each protein was shown as cartoon, with the two switch regions highlighted and labeled.

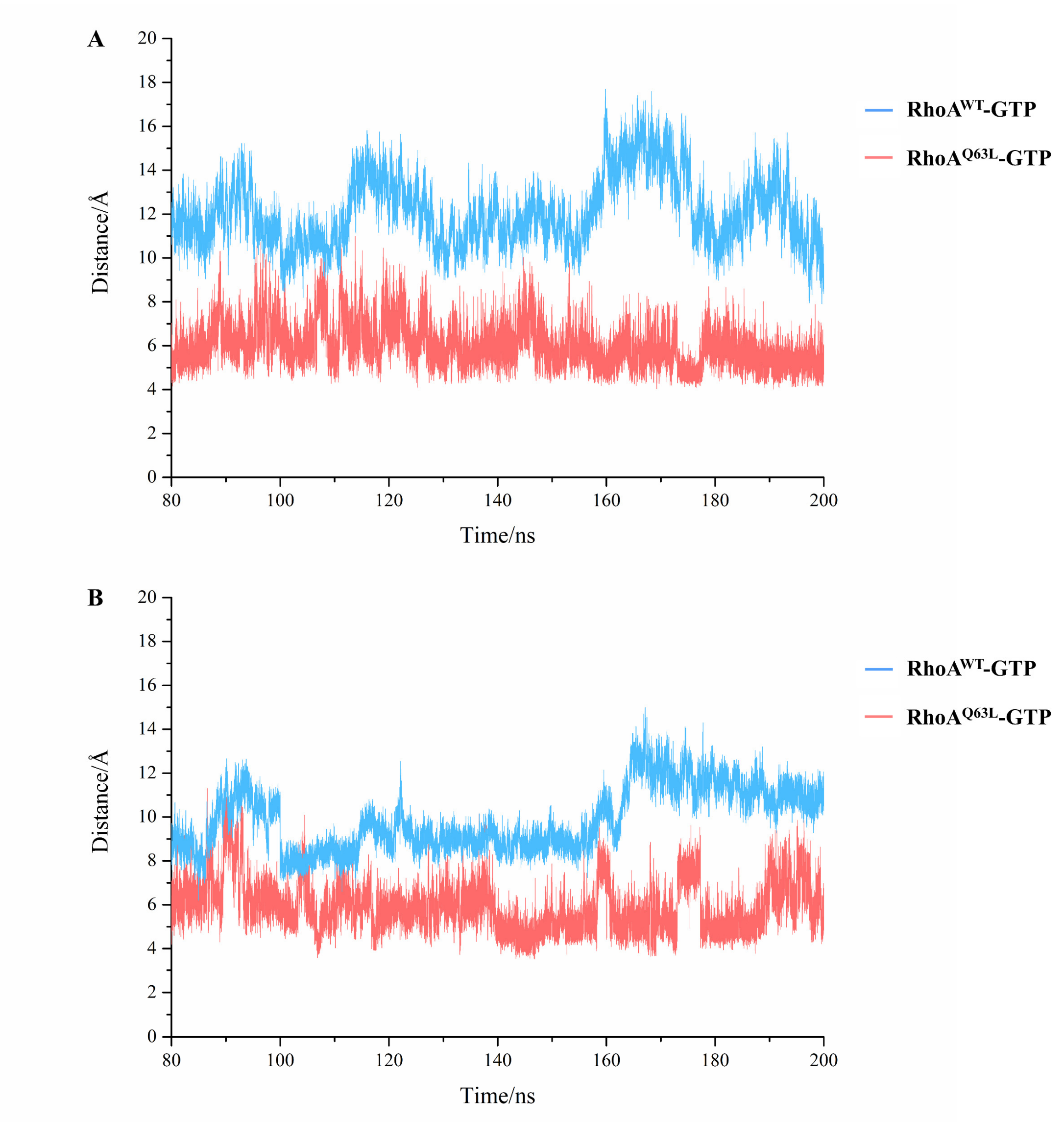

Figure S4. Time series of the distances from the centroid of the side-chain heavy atoms of V38 to the centroid of the side-chain heavy atoms of L63 (or Q63 in the wild type) (A) and to the centroid of the side-chain heavy atoms of Y66 (B) in RhoA^{WT}-GTP and RhoA^{Q63L}-GTP.

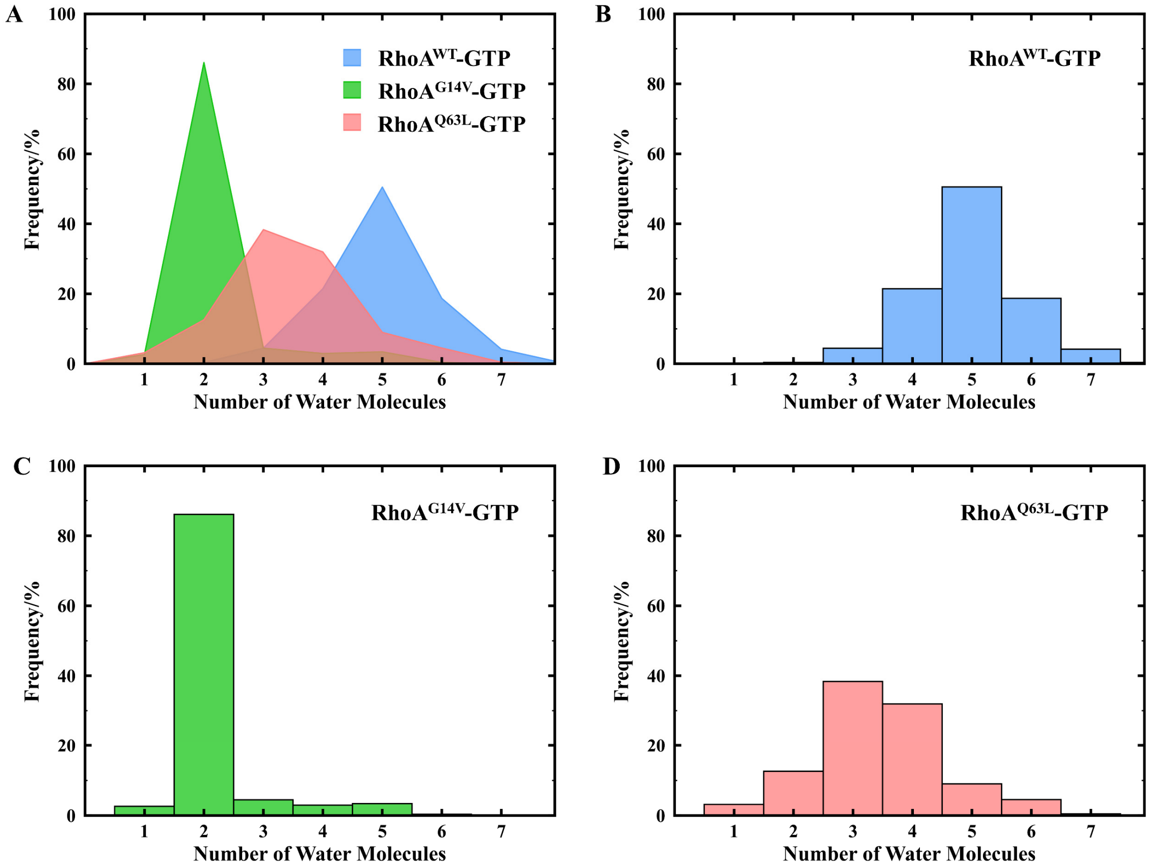

Figure S5. The probability distributions of the average numbers of water molecules within 3.0 Å around the GTP's γ-phosphate in RhoA^{WT}-GTP, RhoA^{G14V}-GTP, and RhoA^{Q63L}-GTP during the simulation time (A) and the individual frequency histograms for each system (B-D).

A

	12–19	32–42	61–78
Human_RhoA	GDGACGKT	EVYVPTVFENY	AGQEDYDRLRPLSYPDTD
Chimpanzee_RhoA	GDGACGKT	EVYVPTVFENY	AGQEDYDRLRPLSYPDTD
Mouse_RhoA	GDGACGKT	EVYVPTVFENY	AGQEDYDRLRPLSYPDTD
Rat_RhoA	GDGACGKT	EVYVPTVFENY	AGQEDYDRLRPLSYPDTD
Zebrafish_RhoA -A	GDGACGKT	EVYVPTVFENY	AGQEDYDRLRPLSYPDTD
Zebrafish_RhoA -B	GDGACGKT	EVYVPTVFENY	AGQEDYDRLRPLSYPDTD
Zebrafish_RhoA -C	GDGACGKT	EVYVPTVFENY	AGQEDYDRLRPLSYPDTD
Zebrafish_RhoA -D	GDGACGKT	EVYVPTVFENY	AGQEDYDRLRPLSYPDTD

B

Species	Length	Identity	Similarity
Human_RhoA	193	/	/
Chimpanzee_RhoA	193	100.0%	100.0%
Mouse_RhoA	193	99.5%	100.0%
Rat_RhoA	193	99.5%	100.0%
Zebrafish_RhoA-A	193	93.3%	96.4%
Zebrafish_RhoA-B	193	95.9%	96.9%
Zebrafish_RhoA-C	193	96.4%	97.9%
Zebrafish_RhoA-D	193	94.3%	97.4%

Figure S6. Comparisons between RhoA encoded in human and other species. (A) Sequence alignment of the P-loop (residues 12-19), switch I (residues 32-42), and switch II (residues 61-78) regions for different RhoA proteins. The two mutation sites G14 and Q63 are colored green and pink, respectively. (B) Sequence similarities between RhoA encoded in human and other species.

Table S1. Hydrogen bonds between GTP and RhoA with an average fraction of more than 75% in any of the three systems over the last 120 ns of the simulations.

**Molecule (Group)**		**System (Fraction%)**		
**GTP**	**RhoA**	**RhoA^{WT}-GTP**	**RhoA^{G14V}-GTP**	**RhoA^{Q63L}-GTP**
GTP@O3G	LYS_18@NZ-HZ	97.23†	99.99†	97.16†
GTP@O2G	PRO_36@CA-HA	9.99	89.02	42.79
GTP@O2G	THR_37@N-H	0.01	98.90	77.77
GTP@O1G	ALA_15@N-H	97.72	99.79	99.05
GTP@O1G	TYR_34@OH-HH	90.47	100.00	99.93
GTP@PB	LYS_18@NZ-HZ	64.52†	81.13†	73.30†
GTP@O2B	THR_19@N-H	95.73	98.73	94.96
GTP@O1B	GLY_17@N-H	97.37	99.39	98.99
GTP@O1B	LYS_18@NZ-HZ	99.98†	99.98†	99.88†
GTP@O1B	LYS_18@CG-HG3	71.09	82.84	77.79
GTP@O1B	LYS_18@N-H	99.98	99.99	99.98
GTP@O3A	GLY_17@N-H	73.81	73.99	75.45
GTP@O1A	CYS_20@N-H	99.92	99.98	99.98
GTP@O1A	CYS_20@SG-HG	98.12	98.43	97.92
GTP@N7	ASN_117@ND2-HD21	95.76	96.88	97.17
GTP@O6	LYS_162@N-H	95.57	96.34	95.06
GTP@O5′	TYR_34@CD1-HD1	40.38	81.99	72.64
GTP@N1-H1	ASP_120@OD1	99.95	97.26	96.40
GTP@N1-H1	ASP_120@OD2	82.34	91.12	89.28
GTP@N2-H21	ASP_120@OD2	99.94	99.88	93.53
**Total Fraction**		**1609.88**	**1885.63**	**1779.02**

†, total fraction of the hydrogen bonds between NZ-HZ1, NZ-HZ2, NZ-HZ3 of Lys18 and O3G, PB, O1B of GTP.
